# Preimplantation Genetic Diagnosis for Myotonic Dystrophy Type 1 and Analysis of the Effect of the Disease on the Reproductive Outcome of the Affected Female Patients

**DOI:** 10.1155/2017/9165363

**Published:** 2017-11-14

**Authors:** Raquel María Fernández, María Dolores Lozano-Arana, Beatriz Sánchez, Ana Peciña, Juan Carlos García-Lozano, Salud Borrego, Guillermo Antiñolo

**Affiliations:** ^1^Department of Genetics, Reproduction and Fetal Medicine, Institute of Biomedicine of Seville (IBIS), University Hospital Virgen del Rocío, CSIC, University of Seville, Seville, Spain; ^2^Centre for Biomedical Network Research on Rare Diseases (CIBERER), Seville, Spain

## Abstract

Myotonic dystrophy type 1 (DM1) is the most common adult muscular dystrophy and presents an autosomal dominant inheritance. A reproductive option for the families affected is preimplantation genetic diagnosis (PGD). One limitation of this option is the nonoptimal response to ovarian stimulation of the women with DM1, although controversial results exist regarding this subject. In this study, we have analyzed the results of the PGD program applied to DM1 at our institution. A total of 35 couples have been included in our program since 2010, and 59 cycles have been performed. The percentage of transfers per cycle was 64.4% and the live birth rate per cycle was 18.6%. Interestingly, statistically significant differences were observed for the clinical results in the group of couples with an affected female versus the group with an affected male or versus a group of couples with different referral reasons. Specifically, both the percentage of mature oocytes out of the total oocytes retrieved and the percentage of fertilization were considerably lower in the group of DM1 females. Our findings would suggest the possibility of achieving less favourable PGD outcomes in women with DM1 in comparison with other pathologies, although the underlying mechanism remains unknown.

## 1. Introduction

Steinert's disease or myotonic dystrophy type 1 (DM1, OMIM#160900) is a multisystem disorder that affects skeletal and smooth muscle as well as the eye, heart, endocrine system, and central nervous system. It is the most common form of adult muscular dystrophy with an overall estimated worldwide prevalence of 1 : 20,000 [1] and an autosomal dominant inheritance. The disease results from an expansion of a CTG trinucleotide repeat in the 3′UTR region of the* DMPK* gene (OMIM*∗*605377), located within the 19q13.32 region.

The clinical findings, which span a continuum from mild to severe, have been categorized into three somewhat overlapping phenotypes (mild, classic, and congenital) that generally correlate with CTG repeat size. In this sense, the CTG repeat is highly polymorphic and relatively stable within the general unaffected population, ranging from 5 to 34 repeats. Alleles from 35 to 49 repeats (premutation alleles) are not associated with any DM1 clinical sign, but are not stable and may expand in length during meiosis resulting in an increased risk for the offspring to inherit repeat lengths longer than those present in the transmitting parent and associated with the disease. In the milder forms of the disease, the patients are almost asymptomatic and carry 50–150 repeats, while classic DM1 patients have 100–1000 repeats and present muscle weakness and wasting, myotonia, cataract, and often cardiac conduction abnormalities. Finally, congenital cases can have >2000 repeats and the disease is characterized by hypotonia and severe generalized weakness at birth, often with intellectual disability, respiratory insufficiency, and early death [2, 3].

Because of the instability of the repeats tract, expansions of CTG repeats can occur during parent-offspring transmission from generation to generation, which are usually associated with an earlier age of onset and increased severity of the disease. This anticipation phenomenon occurs most frequently during maternal transmission, whereas with paternal transmission there is also a possibility of a decrease in the number of repeats [4, 5].

Reproductive options for the couples with familial history of DM1 include prenatal diagnosis followed by possible termination of an affected pregnancy. However, the decision whether or not to terminate is frequently hard and difficult, especially since the prediction of phenotype is not possible because of the overlap of CTG repeat length associated with the three phenotypes and the possibility of somatic mosaicism for the size of the CTG expansion.

An alternative strategy for couples is preimplantation genetic diagnosis (PGD). PGD consists in the genetic analysis of at least one blastomere taken from in vitro fertilized embryos on day 3 at the cleavage stage or at the blastocyst stage. Only unaffected embryos are transferred to the maternal uterus, avoiding the physical and psychological consequences of the termination of pregnancies in the case of affected fetuses detected later by prenatal diagnosis. Nevertheless, the PGD option has also important limitations, particularly in the context of this disease, such as the nonoptimal response to ovarian stimulation of the women with DM1 [6–8]. In fact, opposed to the consensus about male infertility [9, 10], the association between DM1 and female infertility is controversial and further studies would be required to go deeper into this subject [6–8, 11, 12].

Here we present the results of our Program of PGD of DM1. All the procedures were performed at the University Hospital Virgen del Rocío in Seville, Spain (HUVR). In addition, given the heterogeneity in the findings previously reported, we have also analyzed our results through several kinds of comparative studies in order to evaluate the possible association between the disease and the reproductive capacity in the women affected.

## 2. Materials and Methods

### 2.1. Inclusion of Couples in Our PGD Program for DM1, Assisted Reproductive Techniques, and Embryo Biopsy

Since 2010, a total of 39 couples requested their inclusion in our PGD program for the selection of embryos free of DM1. During the first consultation, the couples should provide a clear and accurate genetic test report, confirming the presence of a* DMPK* allele with a number of CTG repeats within the premutation or the full mutation range (≥35 repeats). Then, the general protocol for couples included in the PGD program was followed as previously described [13].

Of the 39 initial couples, 4 were excluded of our program because of serum FSH levels >14 mUI/mL or estradiol levels >60 pg/mL and/or at least two consecutive positive results for the clomiphene citrate challenge test. These are the exclusion criteria followed by our institution as established in the Assisted Human Reproduction Guide for the Andalusian Public Health System. The remaining 35 couples (22 with an affected female and 13 with an affected male, all of them with the classical form of the disease) had at least 1 PGD cycle.

**Figure 1:**
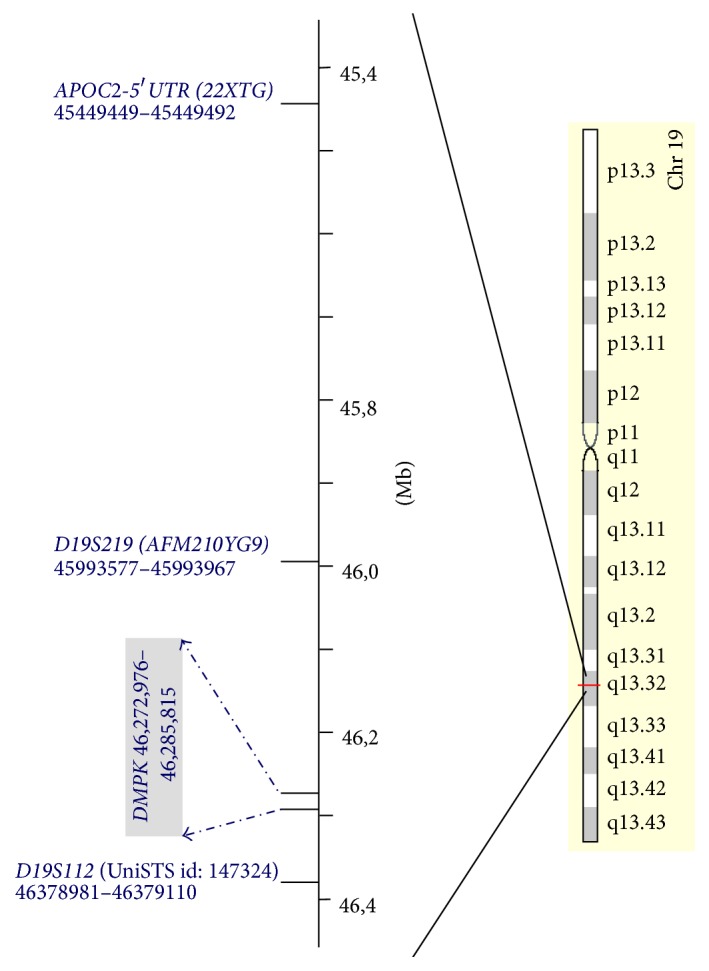
Location of the STR markers used for PGD of DM1.

PGD for DM1 is carried out in our centre by a multiplex PCR method that includes the direct analysis of the CTG repeats number in* DMPK* combined with an indirect analysis using short tandem repeats (STRs) located in the neighbouring regions of this gene [14, 15] (Figure 1). Therefore, once the results of the basic tests meet with the established quality requirements for the assisted reproduction techniques, informative analyses are performed in the context of the couples and their respective families to infer the “disease haplotypes” using such multiplex method.

Controlled ovarian stimulation, oocytes retrieval, and embryos biopsy are performed as previously described [13]. Finally, transfer of up to 2 unaffected embryos is performed on day 5 [13].

### 2.2. Multiplex PCR Protocol

*DMPK* repeat region together with the polymorphic markers* APOC2*,* D19S219,* and* D19S112* was selected to design a multiplex PCR protocol. These markers had been previously used for PGD for DM1 [14, 15] (Figure 1, primer sequences available on request).

A one-step multiplex single-cell fluorescent PCR is used for the simultaneous amplification of the 4 markers, using the QIAGEN® Multiplex PCR kit (QIAGEN, GmbH; Hilden, Germany). The optimized reaction mix contains 0,2 *μ*M each primer, 5x Sol Q, and 2x QIAGEN Multiplex PCR Master Mix, for a final volume of 15 *μ*L. The PCR program is as follows: 15 minutes at 94°C, 10 cycles of 30 seconds at 96°C, 1 minute at 61.0°C, and 1 minute at 72°C, followed by 35 cycles of 30 seconds at 94°C, 1 minute at 61.0°C, and 1 minute at 72°C, and a final extension of 15 minutes at 60°C. PCR products are analyzed on an ABI3730 automated sequencer (Applied Biosystems, Foster City, CA).

### 2.3. Haplotyping of the Embryos

After at least 30 minutes at −80°C, cells are lysed by incubation at 65°C for 10 minutes.

The corresponding genetic analysis of the embryos is subsequently performed using the previously selected combination of markers and the described one-step multiplex fluorescent PCR protocol at the single-cell level.

### 2.4. Statistical Analyses

Several comparative tests were performed to analyze the results obtained in 3 groups of couples: couples with a DM1 affected male, couples with a DM1 affected female, and couples included in the PGD program for a different referral reason. With regard to the third group, we decided to select couples included in the PGD program for hemophilia A, since this group had a similar number of couples to the group of women affected by DM1 and because their data on clinical outcomes were available and had been previously published by our group [13].

Data were analyzed employing the Statistical Package for Social Sciences (SPSS) Version 22.0 for Windows. Statistical significance was calculated using the Chi Squared test with Yates correction, and statistical significance was set at *p* < 0.05.

### 2.5. Ethics Approval and Consent to Participate

Informed consent of all PGD related procedures, such as the use of their clinical outcomes in this study, was signed by the couples. This study was approved by the Ethics Committee for clinical research of the University Hospital Virgen del Rocío (Seville, Spain) and complies with the tenets of the declaration of Helsinki.

## 3. Results

### 3.1. Overall Clinical Results for the PGD Cycles

The overall clinical results for the PGD cycles for DM1 are summarized in Table 1.

A total of 59 cycles were performed for 35 couples in which the males (13 couples, 26 cycles) or the females (22 couples, 33 cycles) were affected of DM1. Worth of note, 9 out of the 59 cycles (15.3%) resulted in no embryos.

The overall fertilization rate, considering the correctly fertilized oocytes out of the total number of mature injected oocytes, was 54.2% (298 out of 560 oocytes). A total of 222 out of the 298 embryos were analyzed (74.5%), with a very variable number of embryos analysed per cycle, ranging from 1 to 11. Finally, 200 out of the 222 analysed embryos (89.6%) were reliably diagnosed. Of them, 92 (46%) were found to carry the parental haplotype linked to the disease, while 108 (54%) were diagnosed as “nonaffected” of DM1.

Finally, 58 embryos were transferred in 38 out of the 59 cycles, which corresponds to a transfer rate of 64.4% (Table 1). Biochemical pregnancy (considered when *β*-hCG values ≥5 UI/l, 9 days after transfer) was achieved in 17 of the 38 cycles (44.7%), for 17 of the 35 couples (48.6%). Pregnancy was subsequently confirmed by echography in 11 cases, leading to clinical pregnancy rate of 18.6% per initiated cycle and of 28.9% per transfer. Finally, birth at term of 11 unaffected children for 10 couples was achieved, which corresponds to a live birth rate of 18.6% per initiated cycle and of 28.9% per transfer.

A total of 12 supernumerary unaffected embryos were cryopreserved for later transfer.

### 3.2. Comparative Analyses of the Clinical Results in Our Institution

Significant differences were observed in the clinical results obtained when comparing the couples with the affected male versus those with the affected female. Of note, 10 out of the 13 DM1 males (76.9%) and just 6 out of the 22 partners of the DM1 females (27.3%) presented an altered seminogram in terms of sperm count, motility, and/or morphology.

Regarding the PGD cycles, 7 out of the 9 cycles that had resulted in no embryos corresponded to couples in which the DM1 patient was the female (21.2%), while for just 2 of the cycles the DM1 patient was the male (7.7%). No differences were observed regarding the total number of oocytes retrieved per cycle in the two groups (Table 1). However, statistically significant differences were observed when comparing the number of mature oocytes out of the total number of oocytes retrieved in both kinds of couples (62.9% versus 71.6%; *χ*^2^ with Yates correction = 6.70, *p* = 0.0096*←*). When comparing this parameter between the total couples affected by DM1 versus couples included in the PGD program because another referral reason such as hemophilia A (HA-couples), differences were observed although not reaching statistical significance (66.8% versus 71.6%; *χ*^2^ with Yates correction = 3.35, *p* = 0.067). Interestingly, if such comparisons are performed between just the couples with the male affected by DM1 versus HA-couples, almost the same results are obtained (71.6% in both groups, *χ*^2^ with Yates correction = 0.01, *p* = 0.9415). However, if comparisons are performed between just the couples with the female affected by DM1 versus HA-couples, statistically significant differences were found (*χ*^2^ with Yates correction = 8.39, *p* = 0.0066*←*) (Table 1).

The difference observed for both groups regarding the percentage of oocytes fertilized is also remarkable (48.9% versus 57.7%; *χ*^2^ with Yates correction = 3.97, *p* = 0.0463*←*). The fact that a total of 15 out of the 33 cycles of the DM1 females (45.5%) just gave ≤2 embryos in contrast with 4 out of the 26 cycles (15.4%) of the DM1 males is quite striking. Moreover, fertilization rate <50% was obtained for 18 out of the 33 cycles of the DM1 females (54.6% of the cycles) and just for 5 out of the 26 cycles of the DM1 males (19.2% of the cycles). When comparing this parameter between the total couples affected by DM1 versus HA-couples, a significant *p* value is again obtained (53.2% versus 64.7%, *χ*^2^ with Yates correction = 12.33, *p* = 0.0004*←*). While such significance disappears when restricting the analysis to the couples with the affected DM1 male versus HA-couples (57.7% versus 64.7%, *χ*^2^ with Yates correction = 3.09, *p* = 0.0789), it increases when performing the analysis between the couples with the affected DM1 female versus HA-couples (48.9% versus 64.7%, *χ*^2^ with Yates correction = 16.57, *p* = 0.00005*←*).

Regarding the percentage of embryos of enough quality to be analyzed, as well as the percentage of embryos that became informative after the molecular analysis by multiplex PCR, the results were quite similar among all the groups analyzed.

Higher values were observed when analyzing the number of cycles reaching a transfer, the number of cycles resulting in a clinical pregnancy, and the number of cycles resulting in a live birth, in the couples with the affected male in comparison with those with the affected female, but such differences were not statistically significant. Because of the different mode of inheritance of DM1 and HA, and the subsequent different theoretical percentage of transferable embryos (50% versus 75%), comparisons among the results of the PGD program for those parameters in these two diseases were not precedent.

On the other hand, comparative analyses of the clinical results for DM1 females undergoing PGD in our institution versus the results in other centres are shown in Table 2. For the other institutions, data were either directly taken or inferred from previous publications [8, 12, 15].

## 4. Discussion

Despite the general inherent drawbacks of PGD such as the need for IVF, cost, and the risk of misdiagnosis, this strategy offers an alternative for the couples who are unwilling to accept prenatal diagnosis leading to possible termination of pregnancies. In the particular case of DM1, the decision to terminate pregnancies is even more difficult given that the extent of disability cannot be efficiently predicted from the size of the expansion. Here we report our experience using PGD for patients who carry a* DMPK* pathogenic expansion, summarizing data of 59 cycles in 35 couples. In general, our results are comparable to those of other centres, thus concluding that PGD is a practical option for affected couples. In 2012, the ESHRE PGD Consortium reported the results of 10 years of data collection from several international PGD centres [16]. In general, a total number of 4733 cycles for monogenic diseases were reported during their first 10 years of data collection leading to a clinical pregnancy rate of 29% per transfer, quite similar to the overall 28.9% achieved in our institution for DM1. Moreover, the PGD for DM1 option has the added value that it indirectly solves the problem of male infertility associated with the disease. Primary hypogonadism, expressed as testicular atrophy, oligospermia, or azoospermia is common among men with the disease [9, 10]. In this sense, we have observed that around three quarter parts of the couples with an affected DM1 male may have decided to opt for assisted reproductive techniques to have offspring because infertility problems.

One of our most interesting findings is that the percentage of mature oocytes retrieved per cycle after ovarian stimulation was considerably lower in the group of couples with a DM1 female than in the other groups, reaching statistical significance. Since no significant differences existed for the median age of the groups compared, this finding would suggest that the presence of expansion of the CTG repeat within the* DMPK* gene is not just related to myotonic, cardiorespiratory, and ophtalmological manifestations, but also to reproductive difficulties in females, thus having an impact in IVF or PGD outcome. In this sense, controversy has been already reported regarding the association between female infertility and DM1. Some authors did not find statistically significant association between the disease and the PGD outcome in DM1 female patients though, as in our case, clinical pregnancy rates and live birth delivery rates were lower for couples with a DM1 female compared with couples with a DM1 male [11]. Other comparative study showed no significant difference concerning the number of oocytes, embryos, and pregnancy rate between DM1 females and controls of *X*-linked disease carriers [12]. In contrast, some other studies reported that women affected by DM had worse response to ovarian stimulation and lower clinical pregnancy rates compared with carriers of *X*-linked disorders [6], or women undergoing Intracytoplasmic Sperm Injection (ICSI) due to male infertility [7]. Moreover, Srebnik et al. [8] published their experience of using PGD for female patients with DM1 in comparison with women with other autosomal dominant or *X*-linked diseases and found statistically significant lower ovarian response to stimulation, decreased embryo quality, and lower clinical pregnancy and live birth rates in the first group. They proposed that the mutant* DMPK* mRNA may accumulate in ovarian granulose cells, in the same way that it occurs with muscle cells, thus producing toxicity and contributing to impaired ovarian function [8].

The ovarian response resulting from controlled ovarian stimulation is associated with a large interindividual variability. Antimüllerian hormone (AMH) and Antral Follicle Count (AFC) are the biomarkers that have provided the best performance in terms of predicting ovarian response to gonadotropins [17]. Comparisons of AMH and/or AFC values in our three groups of patients would have been optimal to analyze a possible impact of the disease in ovarian response, as previously evaluated in other studies [6–8]. Unfortunately, those parameters were just registered in the clinical records of our patients during the last three years, and therefore comparisons were not possible. Nevertheless, since no statistically significant differences were observed regarding the total oocytes retrieved in the three groups, it is not expectable that the ovarian response to stimulation is severely affected by the presence of the* DMPK* expansion. A different mechanism must be underlying the significant differences observed for maturation of oocytes.

On the other hand, the high variability observed in the results and conclusions of the different centres may be due to a combination of several factors such as the different protocols, criteria for inclusion, and patients preselection for the programme. In this line, for instance, we are just considering the cycles with oocyte retrieval out of all the initiated cycles, and therefore cancelled cycles because poor or null ovarian response are not being taken into account.

On the other hand, the difference observed for the percentage of fertilization is also quite interesting, which we found to be significantly lower for the couples with DM1 females, leading also to lower figures for the percentage of transfers per cycle, the percentage of clinical pregnancy, and the live birth rate per cycle in this group. This lower fertilization rate for DM1 females had not been observed in any of the other previous studies [6, 8, 12]. A possible explanation would be that the toxicity produced by the mutant* DMPK* mRNA accumulation may be affecting not only the maturity but also the fertilizability of the oocytes. A correlation may exist between the length of the* DMPK* expansion and the capability of oocytes to fertilize but, unfortunately, the unavailability of the exact and precise number of CTG repeats has not let us analyze the existence of such association.

## 5. Conclusions

In conclusion, our results seem to support a relationship between DM1 disease and a decrease in female fertility, although further investigation is required to identify the responsible mechanisms. It is plausible that the identification of such mechanisms would allow the search for therapeutic measures that could lead to the improvement of the results of PGD in the context of this pathology.

## Figures and Tables

**Table 1:** Clinical data for PGD of DM1 at HUVR.

	Couples with affected female	Couples with affected male	Total	Couples with haemophilia A
Number of couples treated	22	13	35	18
Maternal age	33.2 ± 3.7	32.1 ± 3.8	32.7 ± 3.7	31.8 ± 2.9
Number of cycles performed	33	26	59	29
Number of cycles performed per couple	1.5 ± 0.7	2.0 ± 0.6	1.7 ± 0.7	1.7 ± 0.8
Number of oocytes retrieved	458	380	838	570
Number of oocytes retrieved per cycle	13.9 ± 7.9	14.6 ± 8.5	14.2 ± 8.1	18.4 ± 10.1
Number of mature oocytes submitted to ICSI	288	272	560	408
% of mature oocytes out of total oocytes retrieved	**62.9**%	**71.6**%	**66.8**%	**71.6**%
Number of mature oocytes submitted to ICSI per cycle	8.7 ± 5.8	10.5 ± 5.7	9.5 ± 5.8	13.6 ± 7.3
Number of oocytes fertilized	141	157	298	264
% of oocytes fertilized	**48.9**%	**57.7**%	**53.2**%	**64.7**%
Number of oocytes fertilized per cycle	4.3 ± 3.9	6.3 ± 4.2	5.1 ± 4.1	8.8 ± 5.6
Number of embryos analyzed	108	114	222	205
% of embryos analyzed	76.6%	72.6%	74.5%	77.7%
Number of embryos analyzed per cycle	3.3 ± 3.1	5.1 ± 2.6	3.8 ± 3.1	6.8 ± 4.5
Number of informative embryos	96	104	200	180
% of informative embryos	88.9%	91.2%	89.6%	87.8%
Number of transfers	20	18	38	26
% of transfers	60.6%	69.2%	64.4%	89.7%
Number of embryos transferred	29	29	58	48
Number of embryos transferred per cycle	0.9 ± 0.8	1.1 ± 0.9	1.0 ± 0.8	1.5 ± 0.8
Number of biochemical pregnancies	7	10	17	12
Number of clinical pregnancies	5	6	11	7
% of clinical pregnancies per cycle	15.5%	23.1%	18.6%	24.1%
% of clinical pregnancies per transfer	25.0%	33.3%	28.9%	26.9%
Implantation rate	17.2%	20.7%	19.0%	16.7%
Number of pregnancies went to term	5	5	10	7
Number of babies born	5	6	11	8
Live birth rate per cycle	15.2%	23.1%	18.6%	24.1%

Bold-typed are the parameters that are significantly different when comparing the group of couples with an affected female versus the one with an affected male or versus the group of couples of PGD for haemophilia A.

**Table 2:** Clinical results of PGD cycles for couples with the female affected of DM1 at different centres.

	HUVR, Seville, Spain (this work)	Shaare-Zedek Medical Centre, Jerusalem	UCL Centre for PGD, London, UK	CHU Montpellier, France
Number of couples treated	22	21	13	17
Maternal age	33.2 ± 3.7	30.7 ± 5.2	32.8 ± 3.4	32.1 ± 3.2
Number of cycles performed	33	57	18	33
Number of cycles performed per couple	1.5 ± 0.7	2.71 ± 2.05	1.38 ± 0.51	1.94
Number of mature oocytes submitted to ICSI per cycle	8.7 ± 5.8	7.96 ± 6.6	9.82 ± 5.6	NA
% of oocytes fertilized	49.7%	70.2%	66.5%	NA
% of transfers	60.6%	61.4%	55.5%	42.4%
% of clinical pregnancies per cycle	15.5%	17.5%	22.2%	15.2%
% of clinical pregnancies per transfer	25%	28.57%	40.0%	35.7%
Number of pregnancies went to term	5	8	4	NA
Live birth rate per cycle	15.2%	14.0%	27.8%	NA
Live birth rate per transfer	25%	22.9%	50%	NA

## References

[1] Theadom A., Rodrigues M., Roxburgh R. (2014). Prevalence of muscular dystrophies: a systematic literature review. *Neuroepidemiology*.

[2] International Myotonic Dystrophy Consortium (IDMC) (2000). New nomenclature and DNA testing guidelines for myotonic dystrophy type 1 (DM1). *Neurology*.

[3] Bird T. H. Myotonic Dystrophy Type 1. https://www.ncbi.nlm.nih.gov/books/NBK1165/..

[4] Harper P., Dyken P. (1972). Early-onset dystrophia myotonica evidence supporting a maternal environmental factor. *The Lancet*.

[5] Puymirat J., Giguère Y., Mathieu J., Bouchard J.-P. (2009). Intergenerational contraction of the ctg repeats in 2 families with myotonic dystrophy type 1. *Neurology*.

[6] Feyereisen E., Amar A., Kerbrat V. (2006). Myotonic dystrophy: Does it affect ovarian follicular status and responsiveness to controlled ovarian stimulation?. *Human Reproduction*.

[7] Sahu B., Ozturk O., Deo N., Fordham K., Ranierri M., Serhal P. (2008). Response to controlled ovarian stimulation and oocyte quality in women with myotonic dystrophy type I. *Journal of Assisted Reproduction and Genetics*.

[8] Srebnik N., Margalioth E. J., Rabinowitz R. (2014). Ovarian reserve and PGD treatment outcome in women with myotonic dystrophy. *Reproductive BioMedicine Online*.

[9] Vazquez J. A., Pinies J. A., Martul P., De los Rios A., Gatzambide S., Busturia M. A. (1990). Hypothalamic-pituitary-testicular function in 70 patients with myotonic dystrophy. *Journal of Endocrinological Investigation: Official Journal of the Italian Society of Endocrinology*.

[10] Hortas M. L., Castilla J. A., Gil M. T. (2000). Decreased sperm function of patients with myotonic muscular dystrophy. *Human Reproduction*.

[11] Verpoest W., Seneca S., De Rademaeker M. (2010). The reproductive outcome of female patients with myotonic dystrophy type 1 (DM1) undergoing PGD is not affected by the size of the expanded CTG repeat tract. *Journal of Assisted Reproduction and Genetics*.

[12] Dechanet C., Castelli C., Reyftmann L. (2010). Myotonic dystrophy type 1 and PGD: Ovarian stimulation response and correlation analysis between ovarian reserve and genotype. *Reproductive BioMedicine Online*.

[13] Fernández R. M., Peciña A., Sánchez B. (2015). Experience of preimplantation genetic diagnosis for hemophilia at the university hospital virgen Del Rocío in Spain: technical and clinical overview. *BioMed Research International*.

[14] Dean N. L., Tan S. L., Ao A. (2001). The development of preimplantation genetic diagnosis for myotonic dystrophy using multiplex fluorescent polymerase chain reaction and its clinical application. *Molecular Human Reproduction*.

[15] Kakourou G., Dhanjal S., Mamas T. (2008). Preimplantation genetic diagnosis for myotonic dystrophy type 1 in the UK. *Neuromuscular Disorders*.

[16] Harper J. C., Wilton L., Traeger-Synodinos J. (2012). The ESHRE PGD consortium: 10 years of data collection. *Human Reproduction Update*.

[17] Nelson S. M., Klein B. M., Arce J.-C. (2015). Comparison of antimüllerian hormone levels and antral follicle count as predictor of ovarian response to controlled ovarian stimulation in good-prognosis patients at individual fertility clinics in two multicenter trials. *Fertility and Sterility*.

